# Two cases report on the relationship between white matter hyperintensity volume and cognitive dysfunction in cerebral small vessel disease based on magnetic resonance imaging

**DOI:** 10.1097/MD.0000000000041577

**Published:** 2025-03-07

**Authors:** Yuanyuan Wang, Jingpei Wei, Dayong Ma, Chao Zhang, Haihuan Yang, Ruiyun Yu, Xiaocheng Wang, Li Wang, Linjing Song, Hua Zhang

**Affiliations:** a Beijing University of Chinese Medicine, Beijing, China; b Neurology Department of Dongzhimen Hospital, Beijing University of Chinese Medicine, Beijing, China.

**Keywords:** cerebral small vessel disease, cognitive function, magnetic resonance imaging, white matter hyperintensities.

## Abstract

**Rationale:**

With the development of magnetic resonance imaging (MRI) technology, most of the research tends to find that there is a significant positive correlation between white matter hyperintensities (WMHs) and cognitive dysfunction in cerebral small vessel vascular disease. In this paper, we report 2 cases of cerebral small vessel disease with significant differences in cognitive function and analyze them by multidimensional assessment using imaging technology so as to provide a methodological reference for identifying and diagnosing the causes of differences in cognitive function in cerebral small vessel disease patients.

**Patient concerns:**

Patient 1 was a 64-year-old middle-aged man who presented 10 years ago with slow reaction time, memory loss, and loss of self-care ability, and MRI suggested multiple ischemic infarct foci with cerebral white matter changes. Patient 2 was a 69-year-old middle-aged woman, who did not have any significant abnormalities in cognitive function, and imaging suggested multiple ischemic foci, infarct foci, and cerebral white matter degeneration.

**Diagnosis:**

MRI showed a large fusion of high signal in the cerebral white matter in both patients, which belonged to the category of cerebral small vessel disease according to the Fazekas classification of grade 3.

**Interventions:**

We used imaging techniques to compare the 2 MRI brain white matter high signals in a multidimensional manner and further compared the differences in cognitive functioning between the 2 in terms of brain age, brain functional networks, focal loading of white matter fiber tracts, and neuropsychological scales.

**Outcomes:**

Brain age difference was assessed by whole-brain level and brain function network, white matter fiber bundle lesion load, and Montreal Cognitive Assessment and Mini-Mental State Examination scale scores; the results suggested that patient 1 had relatively poor cognitive function.

**Lessons:**

In this paper, we concluded that the volume of high white matter signal in WMH is not positively correlated with the severity of cognitive impairment. In addition to cerebral WMHs, we believe that alterations in cerebral network connectivity and white matter microstructure may be the neuroimaging basis of cognitive decline in patients with WMH, which may provide a new idea for the early diagnosis of cognitive function in patients with cerebral small vessel disease.

## 1. Introduction

Cerebral small vessel disease (CSVD) refers to the abnormality of cerebral arterioles,^[1,2]^ which mainly affects the supply of white matter of the brain and perforator small arteries and veins, capillaries, and other deep structural vessels, and is a more common chronic progressive vascular disease. Cerebral small vessels play an important role in regulating cerebral blood flow and the blood-brain barrier, and their dysfunction will lead to different degrees of brain tissue damage, ultimately affecting the cognitive function of patients.^[3,4]^ With the acceleration of the aging social process worldwide, the high prevalence of CSVD and cognitive dysfunction caused by it have attracted more and more attention from clinicians and researchers at home and abroad. The clinical manifestations of CSVD at the initial stage are concealed and slowly progress,^[5]^ and its prevalence is highly correlated with age, which is difficult to attract enough attention from patients. Neuroimaging features of CSVD include^[6]^ recent small subcortical infarcts, lacune of presumed vascular origin, white matter hyperintensities (WMHs) of presumed vascular origin, pervascular space atrophy, cerebral microbleed (CMB), iron deposition on the cortical surface (brain siderosis), brain atrophy (brain summary score), small vessel disease composite score (CSVD score), and cerebral cortical microinfarction.

WMHs are dynamic processes in which varying degrees of pathological changes occur in the white matter, and the prevalence of WMHs can rise from 50% to 95% in people aged 45 to 80 years,^[7]^ usually progressing gradually with age and is also known as age-related white matter lesions. WMHs represent lesions such as demyelination, axonal loss, and gliosis. Studies have shown that WMHs are significantly associated with cognitive decline, and magnetic resonance imaging (MRI) multisequence scanning can be used to accurately and agilely identify the number of WMHs at different sites so as to analyze white matter lesions. Vascular cognitive impairment is a large group of clinical syndromes of different severity from mild cognitive impairment (MCI) to vascular dementia (VaD) caused by cardiovascular and cerebrovascular risk factors, significant or insignificant cerebral vessels, resulting in a comprehensive decrease in the quality of daily life of patients and impairment of social activities. CSVD is one of the most common causes of vascular cognitive impairment. Cross-sectional studies^[8]^ have found that the larger the size of vasogenic WMH, the worse the overall cognitive function, particularly the impaired speed of information processing and executive function. MRI is now widely used in the diagnosis of neurodegenerative diseases, and clinical diagnosis of CSVD relies primarily on MRI examinations,^[4]^ which present as hyperintensities or vasogenic lacunae that have been shown to predict increased risk of dementia.^[9]^ However, this association between WMHs and cognitive decline raises the question of whether the magnitude of WMH volume on MRI directly reflects the degree of cognitive impairment.

Current studies on the association of WMHs on MRI in patients with cognitive dysfunction in CSVD are inconclusive. In this paper, we compared 2 cases of CSVD with WMH on MRI and significant differences in clinical cognitive function based on MRI techniques in different dimensions in order to gain some understanding and attention to this difference in clinical practice.

## 2. Case presentation

### 2.1. Case 1

Patient 1: Wang was a 62-year-old male. Eight years ago, the patient presented to our hospital with unresponsiveness and memory loss due to long-term exertion, accompanied by slurred speech, walking to the right deviation, and gradually decreased self-care ability in daily life. Cranial MRI revealed “cerebral infarction.” The patient came to our hospital about every year and was diagnosed with VaD. The symptoms could be temporarily improved after oral western medicine treatment for antiplatelet aggregation, lipid-lowering and plaque stabilization, antihypertensive, and cognitive improvement, which gradually worsened in several years. Five years ago, his family found that the patient had poor spirit, did not want to communicate with others, and developed unstable walking, occasional falls, and fecal and urinary incontinence. In the past week, his family found that the patient had no obvious inducement, apathy, aggravated symptoms, weakness of both lower limbs, aggravated feeling, difficulty in walking, and then accompanied him to the hospital.

The patient had a medical history of hypertension for more than 20 years and type 2 diabetes for 6 years. At present, the blood pressure and blood glucose control are relatively stable, without a special history of trauma or surgery, smoking, or drinking. The admission specialist examination showed clear consciousness, lack of energy, unresponsiveness; decreased calculation and memory, decreased temporal and spatial orientation, impaired expression, decreased writing and repetition ability, and fair listening comprehension; no abnormalities in sensory symmetry, and less stable finger-nose test; and right palmar-jaw reflex (+), right Hoffmann sign (+), bilateral Babinski sign (+), Oppenheim sign (+), and Chadock sign (+). The rest showed no significant abnormality. Repeated cranial MRI and diffusion-weighted imaging (DWI) showed multiple ischemic infarcts and partial softening in the bilateral frontoparietal lobes, centrum semiovale, paraventricular, basal ganglia, thalamus, brainstem, and corpus callosum, with white matter changes; and senile brain changes. The results of assessing the patient’s cognitive function showed Mini-Mental State Examination (MMSE) of 19 and Montreal Cognitive Assessment (MoCA) of 13.

### 2.2. Case 2

Patient 2: Yang was a 69-year-old female who presented to the hospital with unfavorable movement of the right limb, difficulty in lifting, weakened touch sensation, insufficient holding strength of the right hand without obvious inducement 3 days ago, and gradually aggravated subjective symptoms, which were later accompanied by her family members. The patient had a past medical history of hypertension for more than 20 years and regularly took Amlodipine Tablets 10 mg qd to control blood pressure. At present, the blood pressure control was stable; the patient denied any history of diabetes, cerebral hemorrhage, heart disease, or other chronic diseases; and denied any history of surgery or trauma. No history of smoking or alcohol consumption. The admission examination showed body temperature of 36.5°C, a pulse of 78 beats per minute, a respiratory rate of 19 breaths per minute, and a blood pressure of 142/94 mmHg. Other examinations showed no significant abnormalities. Neurological examination showed clear consciousness, fine mental state, fluent speech; right limb weakened, tendon reflexes were symmetrical; and bilateral pathological signs (−). Remaining physical examinations showed no significant abnormalities. Routine laboratory tests on admission were unremarkable, and cranial MRI and DWI showed acute or subacute cerebral infarction beside the left lateral ventricle; multiple cerebral ischemia and infarcts in the bilateral frontoparietal lobe, insula, and centrum semiovale beside the lateral ventricle, basal ganglia, left thalamus, and brainstem; and senile brain changes and white matter degeneration. Neuropsychological assessment showed MMSE of 28 points and MoCA of 24 points.

## 3. Methods

### 3.1. Data processing methods

WMHs and lacunar foci were animated on fluid attenuated inversion recovery (FLAIR) images using MRIcroGL, version 1.2.2, in this paper. T1, T2, and FLAIR images were then analyzed by ANTs, version 2.2.0 (https://github.com/ANTsX/ANTs), and the software package was nonlinearly registered with the Montreal Neurological Institute (MNI)152 template to convert the deformation files obtained by WMH using registration into MNI152 space using the nearest neighbor method, and no spatial transformation was performed because the contour of the lacunar lesion was not obvious after registration. Second, the volumes of MNI space WMH and original space lacunae were counted using Python’s nibabel package, and the large arteries of magnetic resonance angiography (MRA) images were labeled and reconstructed using Slicer version 5.6.1. In addition, in this paper, the DenseNet-based deep learning method and pretraining model of brain age provided by Wood et al^[10]^ were used to evaluate the whole brain age of 2 patients, and the brain age distribution and median of voxels within the range of each brain network were assessed using the currently widely used resting-state 7 large-scale brain network templates,^[11]^ and the voxel-based brain age assessment was performed by referring to the U-Net-based voxel-based brain age calculation method and model proposed by Porescu et al.^[12]^ WMH lesion load and weighted lesion load for deep white matter fibers were calculated as follows: using the white matter fiber probability template provided by Warrington et al^[13]^ (probability threshold of 0.5%; Fig. 10), each voxel’s value was the probability of white matter fiber tract emergence; the 3 axes in space, up and down, front and back, and left and right, intersect the plane perpendicular to these 3 axes with the fiber template, and the plane with the average probability of intersecting voxels reaches the maximum, and its corresponding axis is the main direction of the fiber, and the above analysis is completed using the Python code; and using the analysis method of lesion load and weighted lesion load proposed by Zhu et al,^[14]^ the lesion load and weighted lesion load were calculated for the deep white matter fiber tracts of WMH in 3 directions.

**Figure 1.**
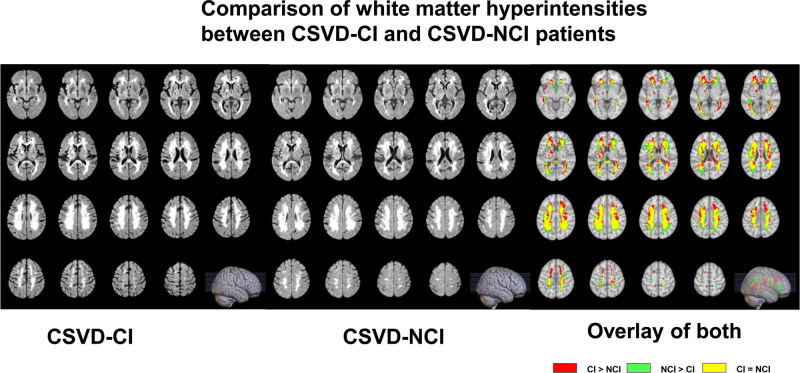
Comparison of white matter hyperintensities between cerebral small vessel disease (CSVD)-CI and CSVD-NCI patients (CSVD-CI indicates patient 1 and CSVD-NCI indicates patient 2). In both cases, the magnetic resonance imaging (MRI) showed a large fusion of cerebral white matter high signals, which were classified as grade 3 according to the Fazekas classification. Comparison of patient 1 and patient 2 showed that there were more overlapping areas of cerebral white matter high signal, and by superimposing MRI, it could be seen that patient 1’s cerebral white matter high signal was higher than that of patient 2, which was concentrated in the bilateral frontal-parietal lobes, the corpus callosum, the parietal ventricles, and the thalamus, while the cerebral white matter high signal of patient 2 was higher than that of patient 1, which was concentrated in the subcortical area. CI = cognitive impairment, NCI = noncognitive impairment.

**Figure 2.**
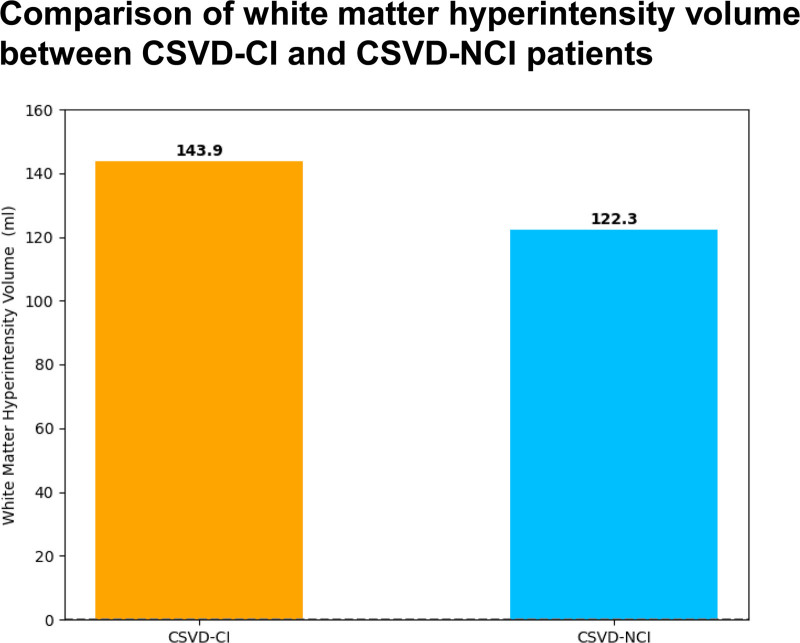
Comparison of white matter hyperintensity volume between cerebral small vessel disease (CSVD)-CI and CSVD-NCI patients. CI = cognitive impairment, NCI = noncognitive impairment.

**Figure 3.**
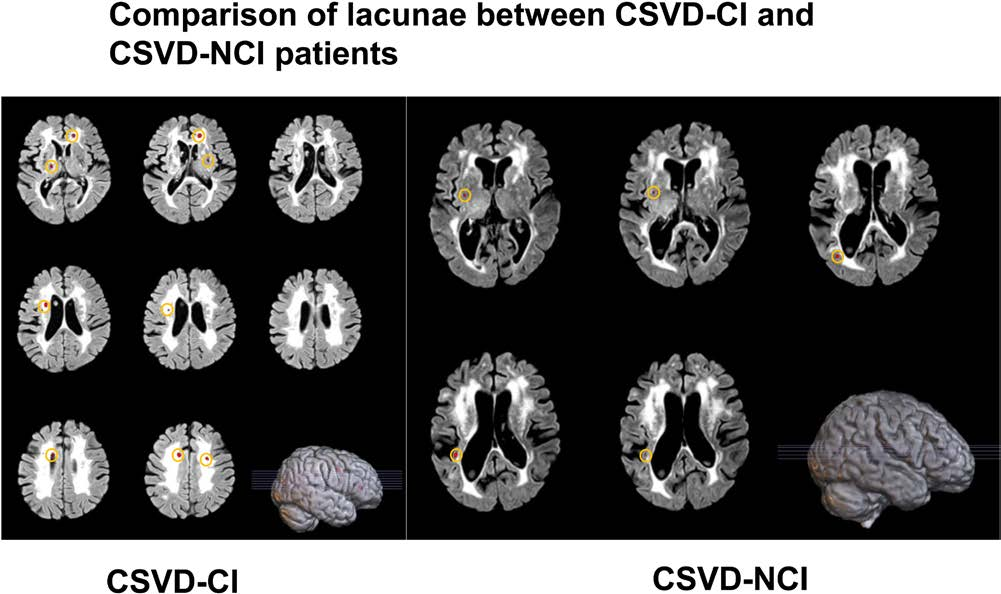
Comparison of lacunae between cerebral small vessel disease (CSVD)-CI and CSVD-NCI patients. CI = cognitive impairment, NCI = noncognitive impairment.

**Figure 4.**
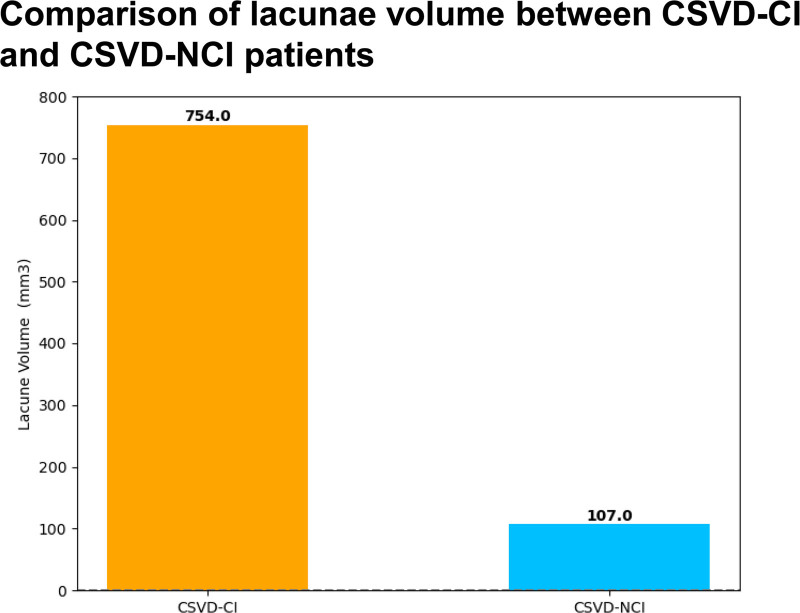
Comparison of lacunae volume between cerebral small vessel disease (CSVD)-CI and CSVD-NCI patients. CI = cognitive impairment, NCI = noncognitive impairment.

**Figure 5.**
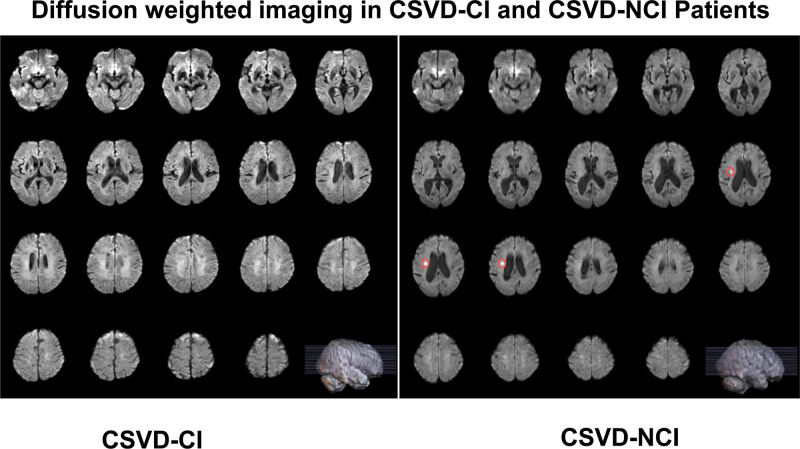
Diffusion-weighted imaging in cerebral small vessel disease (CSVD)-CI and CSVD-NCI patients. The red markers indicate the areas of high signal in the white matter of patient 2. CI = cognitive impairment, NCI = noncognitive impairment.

**Figure 6.**
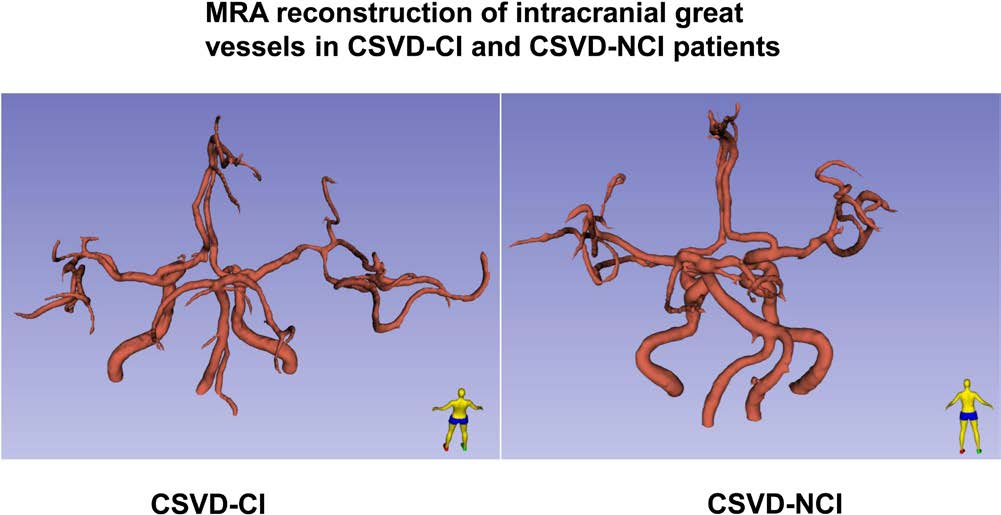
Magnetic resonance angiography (MRA) reconstruction of intracranial great vessels in cerebral small vessel disease (CSVD)-CI and CSVD-NCI patients. CI = cognitive impairment, NCI = noncognitive impairment.

**Figure 7.**
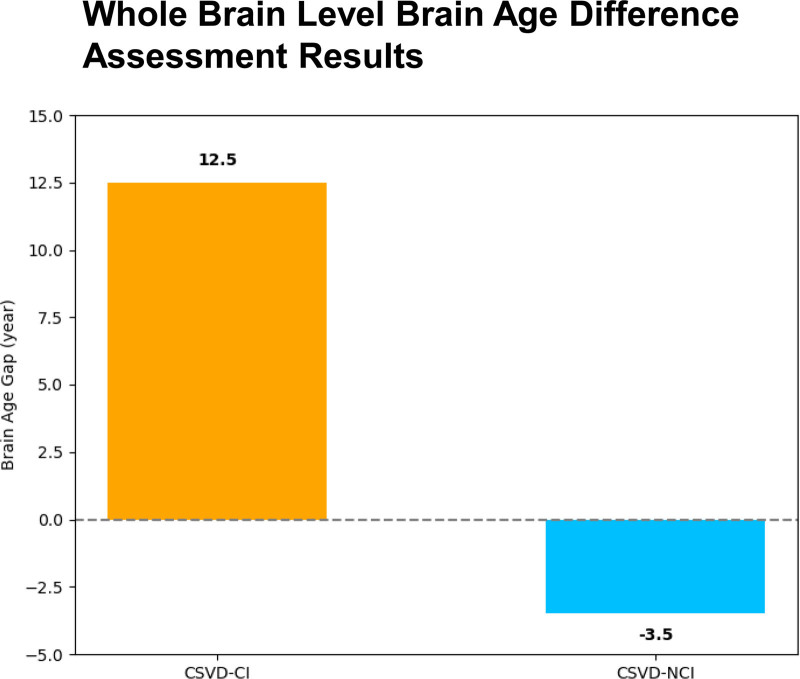
Results of the assessment of brain age differences at the whole-brain level. CSVD = cerebral small vessel disease. CI = cognitive impairment, NCI = noncognitive impairment.

**Figure 8.**
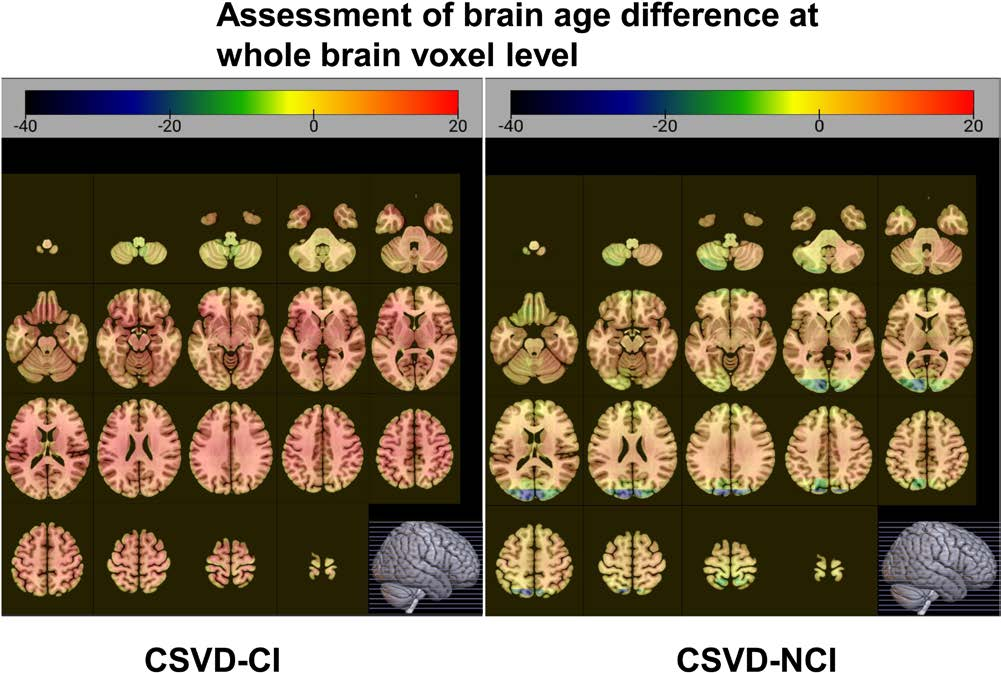
Assessment of brain age difference at whole brain voxel level. CSVD = cerebral small vessel disease. CI = cognitive impairment, NCI = noncognitive impairment.

**Figure 9.**
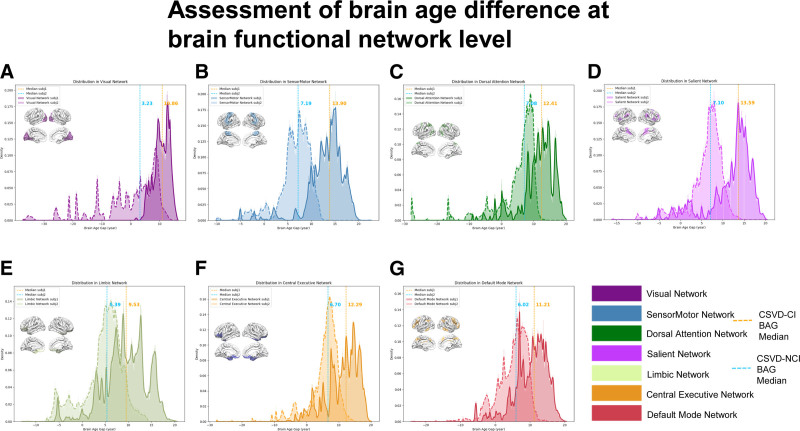
Assessment of brain age difference at brain functional network level. (A) Distribution in visual network. (B) Distribution in sensor motor network. (C) Distribution in dorsal attention network. (D) Distribution in salient network. (E) Distribution in limbic network. (F) Distribution in central executive network. (G) Distribution in default mode network. CSVD = cerebral small vessel disease. BAG = brain age gap, CI = cognitive impairment, NCI = noncognitive impairment.

### 3.2. MRI scan parameters

T1 parameters: repetition time (TR) = 350 ms, echo time (TE) = 2.48 ms, matrix = 580 × 640, field of view (FoV) = 220 × 240 mm, slice thickness = 6.0 mm.

T2 parameters: TR = 6000 ms, TE = 120 ms, matrix = 580 × 640, FoV = 220 × 240 mm, slice thickness = 6.0 mm.

FLAIR parameters: TR = 7800 ms, TE = 89 ms, matrix = 204 × 256, FoV = 204 × 256 mm, slice thickness = 3.6 mm.

DWI parameters: TR = 5400 ms, TE = 100 ms, matrix = 150 × 150, FoV = 240 × 240 mm, slice thickness = 6.0 mm, b value of 1000 s/mm^2^.

MRA parameters: TR = 22 ms, TE = 3.6 ms, matrix = 612 × 768, FoV = 200 × 250 mm, slice thickness = 0.67 mm.

## 4. Outcomes

According to the statistical analysis of imaging findings and WMH volume (Figs. 1 and 2), combined with the clinical symptoms that the severity of cognitive dysfunction in patient 1 was higher than that in patient 2, it was considered that the difference in cognitive dysfunction between the 2 was related to the location of WMHs. In addition, the lacunar volume of patient 1 was significantly higher than that of patient 2, and most of the lacunae of patient 1 were located in the bilateral frontoparietal lobes, centrum semiovale, paraventricular, basal ganglia, thalamus, brainstem, and corpus callosum; patient 2 had scattered external capsules, paraventricular posterior horn of the lateral ventricles, and parieto-occipital junction (Fig. 3). A comparison of the volume of vasogenic lacunae between the 2 patients showed that patient 1 had a significantly larger volume than patient 2 (Fig. 4). Figure 5 shows DWI of 2 patients. The red mark indicates that patient 2 has punctate hyperintensities in the left lateral ventricle, while patient 1 shows periventricular WMHs. Considering the clinical manifestations of the patients, it is likely that the volume, shape, and spatial position of WMHs have significant neuropathological and clinical correlations with cognitive dysfunction. Patient 1 developed a poor mental state, apathy, and reluctance to communicate with others 5 years ago, combined with an MRI showing multiple scattered lacunae in the brain, which was consistent with lacunar infarction mainly manifested as executive dysfunction, significantly reduced motor and processing speed, impaired memory ability, and possible apathy. In addition, MRA reconstruction of the intracranial great vessels in both patients showed that the great vessels were in good condition without significant injury or occlusion (Fig. 6).

Through the assessment of brain age difference at the whole brain level (Fig. 7), the predicted brain age of patient 1 increased by 12.5 years compared with the actual brain age, while the brain age of patient 2 decreased by 3.5 years compared with the actual brain age. Combined with the education level of the 2 patients, patient 1 had a junior high school education, and patient 2 had a college education. According to the results of previous studies, we speculated that the difference in brain age between the 2 patients may be related to education. The difference in brain age between the 2 patients was assessed based on voxel-wise levels, and a positive value indicated an increase in predicted brain age compared to the actual brain age value in the healthy aging model for the corresponding age. As shown in Figure 8, the predicted brain age difference of patient 2 was lower, while the predicted brain age difference of patient 1 was significantly increased. Second, the brain age difference between the 2 patients was assessed based on the functional brain network level (Fig. 9), and all 7 networks showed a significant increase in the predicted brain age difference between patient 1 and patient 2 as a whole compared with the median of the regional brain age difference between patients. In addition, the 2 patients had the highest level of brain age difference in the sensorimotor network; that is, the predicted brain age difference was 13.90 in patient 1 and 7.19 in patient 2, while the brain age difference between the 2 was relatively low in the marginal and visual networks, showing that the predicted brain age difference was 9.53 in the marginal network in patient 1 and 3.23 in patient 2 in the visual network, and the region with the highest brain age difference was located in the sensorimotor network in patient 1, while the most increased in patient 2 was located in the marginal network. It is worth noting that the difference between voxel-based brain age and whole-brain level-based assessment of brain age is due to the fact that they use different deep learning models.

**Figure 10.**
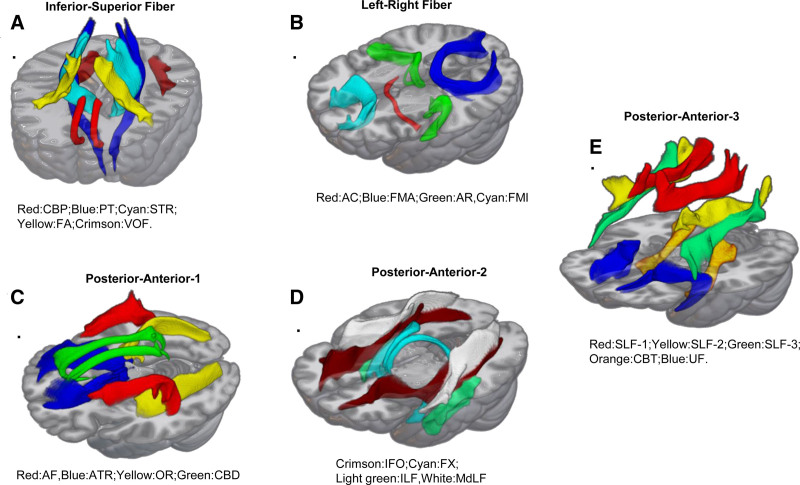
Measurement of deep white matter fibers in different orientations of the lesion load. (A) Inferior-superior fiber: CBP = cingulum subsection: peri-genual, FA = frontal aslant tract, PT = pyramidal tract, STR = superior thalamic radiation, VOF = vertical occipital fasciculus. (B) Left-right fiber: AC = anterior commissure, AR = acoustic radiation, FMA = forceps major, FMI = forceps minor. (C) Posterior-anterior-1: AF = arcuate fasciculus, ATR = anterior thalamic radiation, CBD = cingulum subsection: dorsal, OR = optic radiation. (D) Posterior-anterior-2: FX = fornix, IFO = inferior fronto-occipital fasciculus, ILF = inferior longitudinal fasciculus, MdLF = middle longitudinal fasciculus. (E) Posterior-anterior-3: CBT = cingulum subsection: temporal, SLF-1 = superior longitudinal fasciculus I, SLF-2 = superior longitudinal dasciculus II, SLF3 = superior longitudinal fasciculus III, UF = uncinate fasciculus.

**Figure 11.**
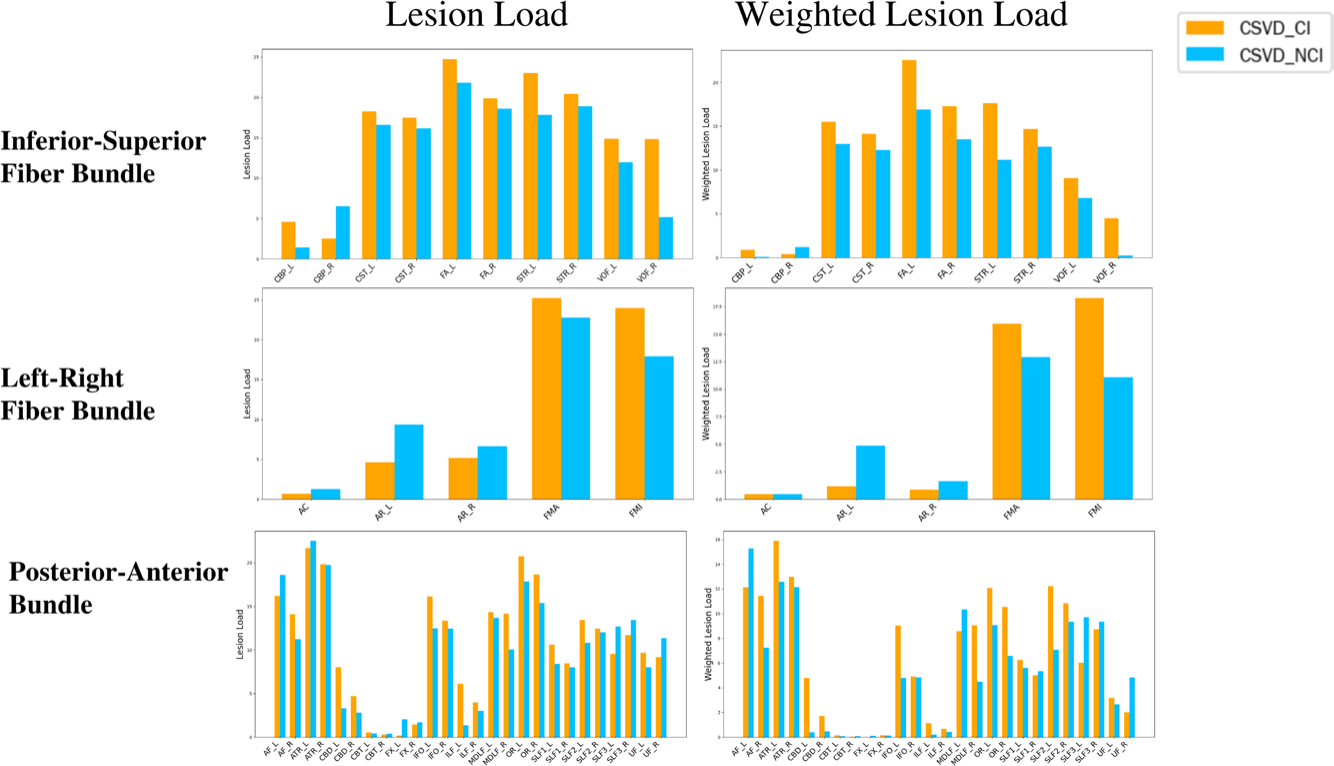
Focal loads and weighted focal loads of the major intracranial white matter fiber tracts. (A) Raw lesion load vs inferior-superior fiber bundle. (B) Raw lesion load vs left-right fiber bundle. (C) Raw lesion load vs posterior-anterior bundle. (D) Weighted lesion load vs inferior-superior fiber bundle. (E) Weighted lesion load vs left-right fiber bundle. (F) Weighted lesion load vs posterior-anterior bundle. The up and down fiber tracts, left and right, and anterior and posterior fiber tracts show that patient 1 has a higher focal load than patient 2 in general, especially in the left frontal oblique fasciculus, the large callosal pincer, and the left anterior thalamic radiation. In terms of primary focal load, the white matter fiber tracts of patient 1 that were significantly higher than those of patient 2 were mainly concentrated in the right occipital longitudinal fasciculus, the small forceps of the corpus callosum, the left dorsal cingulate fasciculus, and the left inferior longitudinal fasciculus, whereas the focal loads of patient 2 that were higher than those of patient 1 were distributed in the right anterior cingulate fasciculus, the left auditory emanation, and the left superior longitudinal fasciculus III. CI = cognitive impairment, CSVD = cerebral small vessel disease, NCI = noncognitive impairment.

In addition, this article shows the relationship between white matter fibers and lesions by comparing and visualizing lesion loads and weighted lesion loads in different directions of deep white matter fiber tracts (Figs. 10 and 11). We also found that there was no significant difference in the original lesion load between the bilateral oblique frontal fasciculus in the ascending and descending fibers, the small forceps of the corpus callosum in the left and right fibers, the bilateral anterior thalamic radiation in the anterior and posterior fibers, the left inferior fronto-occipital fasciculus, and the left superior longitudinal fasciculus II, while there was a significant difference considering the weighted lesion load of fiber direction and cross-sectional fiber density, and it was obvious that the bilateral pyramidal tract weighted lesion load in the ascending and descending fibers was greater in the former. Referring to probabilistic models in previous studies,^[14]^ motion impairment was quantified based on the geometry of the bundle, and weighted lesion loads proved to be more predictive of motion impairment than original lesion loads. Similarly, we found that the region with the highest brain age difference in patient 1 was located in the sensorimotor network, while patient 2 had the most increase in the limbic network, both of which could explain the more severe motor impairment in patient 1, based on brain functional network levels. At the same time, combined with patient 1’s occasional walking difficulty, with decreased self-care ability in daily life and other related clinical manifestations, it can be confirmed that there is a close relationship between white matter fiber lesion load and lesions.

**Figure 12.**
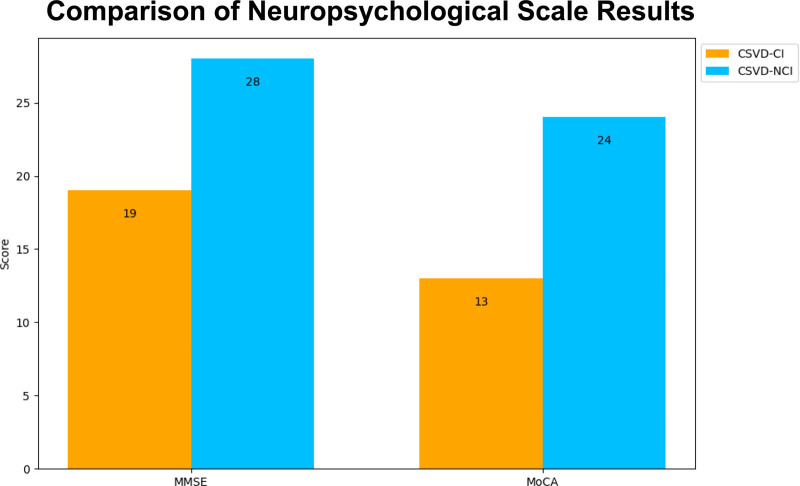
Comparison of neuropsychological scale result. CI = cognitive impairment, CSVD = cerebral small vessel disease, MMSE = Mini-Mental State Examination, MoCA = Montreal Cognitive Assessment, NCI = noncognitive impairment.

Finally, the MMSE and MoCA scores of the 2 patients were statistically analyzed (Fig. 12). The scores of patient 1 were lower, indicating that their cognitive ability was worse than that of patient 2. The MMSE is currently the most widely used cognitive screening scale, and the subitems include time orientation, place orientation, immediate memory, attention and calculation, delayed memory, language and visuospatial, and the analysis index is the total score. Patient 1 scored 19 on the MMSE scale and was in moderate dementia; patient 2 scored 28 and was in normal condition. The cognitive function domains assessed by the MoCA scale mainly included orientation, memory, attention, execution, calculation, visuospatial and abstract thinking, and the MoCA score of patient 1 shown in the figure was significantly lower than that of patient 2.

## 5. Discussion

Multiple mechanisms are involved in the development of CSVD, including hypertension, vascular endothelial cell injury due to vascular inflammation, smooth muscle hyperplasia, thickening of the basement membrane of small vessels, fibroplasia of connective tissue, enlargement of the perivascular space, and vascular endothelial dysfunction due to impaired blood-brain barrier.^[15]^ Because the clinical manifestations of CSVD lack specificity, the diagnosis mainly relies on imaging examination, and its MRI can show recent subcortical small infarcts, lacunae, WMH, enlarged perivascular spaces, or CMBs. Its cognitive function is insidious and atypical in the early stage of the disease, and most patients show significant symptoms of cognitive dysfunction until the middle and late stages of the disease, when some patients have lost their activities of daily living or even progressed to dementia, bringing great economic pressure and care burden to society and patients’ families. The mechanism by which CSVD is associated with cognitive dysfunction is currently not clear and is generally considered to be due to cerebral hypoperfusion, reduced cerebrovascular reactivity, or disruption of the integrity of the blood-brain barrier^[16–18]^; some scholars believe that it is associated with lymphatic system dysfunction.^[19]^ However, it is currently more likely to be associated with pathological changes such as white matter lesions, multiple lacunae, microhemorrhages in the basal ganglia, and perivascular spaces. Second, most studies have attributed the development of cognitive impairment due to CSVD to damage to white matter nerve fibers in cortical-cortical or cortical-ubcortical critical locations, resulting in dysfunction of the neurovascular unit.^[20]^

In this paper, MRI showed WMH in both patients with CSVD, but the distribution sites of WMH were different. In a study of 668 patients with WMH,^[21]^ 39% of participants showed significant WMH progression through follow-up MRI and neuropsychological testing after 3 years, and it was found that patients with periventricular WMH progression had decreased information processing speed and general cognitive ability, while patients with deep WMH progression had no significant change in cognitive function. Papma et al^[22]^ showed that MCI patients with CSVD had more pronounced callosal, internal and external capsule, and periventricular white matter damage than MCI patients without CSVD. Combined with the above findings, it was approximately the same as the results of WMH distribution on MRI in both patients in this paper. Overall, patient 1’s lesion load of white matter fiber tracts was higher than in patient 2, which was also considered to have some correlation with cognitive function differences. Second, in terms of vasogenic lacunae, some scholars believe that smoking causes cerebral ischemia and hypoxia, resulting in lacunar infarction, and point out that the incidence of cognitive impairment in lacunar infarction in smokers is 2.14 times that in lacunar infarction in nonsmokers,^[23]^ but this report showed that both patients had no history of smoking or drinking, so this view could not be verified and supported for the time being.

At present, most studies have confirmed that the predicted brain age can be used as a biomarker of brain aging, and an accurate brain age prediction model and data analysis can provide help for clinical disease diagnosis, which has important clinical significance. In order to further show the differences in brain function and structure between the 2 cases, this report is based on MRI technology and assessed the whole brain age of the patients through 2 dimensions: voxel and brain function network levels. Combined with previous studies, we found that the brain age differences may be related to education. In addition, some studies have predicted neurodegenerative diseases by brain age, which helps early detection of cognitive decline and provides the basis for early clinical intervention and treatment.^[24,25]^ Among them, Cole et al^[26]^ found that the higher the difference in predicted brain age, the worse the original cognitive ability; Gonneaud et al^[27]^ calculated data from 1340 cognitively normal participants based on the predicted brain age model and applied the prediction model to preclinical Alzheimer disease and found signs of accelerated aging in brain structures in patients with MCI and Alzheimer disease; Pardoe et al^[28]^ showed that medically intractable focal epilepsy was associated with structural brain changes similar to premature brain aging. Other studies^[29]^ have also reported significant associations of obesity and high body fat percentage with predicted brain age differences (approximately 10 years). Therefore, brain age prediction is of great value in the diagnosis of diseases as well as early warning interventions. Beheshti et al^[30]^ found that higher predicted brain age differences reflected in different data were significantly associated with decreased MMSE scores. Combined with the results of the 2 cases of brain age difference assessment, it is consistent with the results of this study, and it also suggests that predicting brain age difference values is expected to be an effective marker for neuropsychological screening of cognitive impairment.

WMH is one of the most common MRI features of CSVD. Studies have shown that severe white matter lesions can lead to cognitive decline and induce VaD, depression, gait disorders, etc, of which cognitive dysfunction is mainly manifested as executive and attention decline, while memory is relatively preserved,^[31,32]^ but the degree of cognitive decline is not positively correlated with the severity of WMH, while age, CMBs, multiple basal ganglia perivascular spaces, and education are closely related to cognitive decline.^[33]^ Furthermore, little is currently known about the mechanisms involved in the progression of white matter hyperintense volume. Higher blood pressure control and risk factor management have been shown to slow WMH volume gain and reverse white matter hypersignaling-related brain damage to some extent in elderly hypertensive patients,^[34]^ but the specific mechanism of this WMH presentation has not yet been clarified.

In a population-based longitudinal study that found dynamic changes at white matter hyperintense volumes,^[35]^ Wardlaw et al^[36]^ noted that dynamic changes in white matter hyperintense volumes affected memory function. In recent years, relevant studies^[37–39]^ have found heterogeneity in the relationship between WMH and cognitive decline; that is, the severity of WMH does not match the degree of cognitive decline, and there are contradictions in the results reported in much relevant literature. For example, a meta-analysis study showed that WMH volume was positively correlated with the degree of cognitive dysfunction, and the risk of cognitive dysfunction in patients with CSVD increased with increasing WMH volume^[40]^; Rist et al^[41]^ indicated that patients with greater white matter hyperintense volume impairment had lower cognitive function, while Frison et al^[42]^ did not balance the severity of WMH with the severity of cognitive decline. The WMHs on MRI in both patients reported here were at Fazekas visual scale grade 3, but cognitive function showed significant differences, and there is still a lack of strong evidence on whether the size of WMH volume is positively correlated with cognitive dysfunction, and the correlation between the 2 remains to be further studied.

## 6. Conclusion

In summary, based on MRI, the relationship between WMH volume and cognitive dysfunction in 2 patients with CSVD was reported and analyzed. Through multidimensional comparison of MRI and data processing analysis, it was considered that there was no significant positive correlation between WMH volume and the severity of cognitive dysfunction in MRI, and it also provided imaging technical support for further clinical differentiation of cognitive dysfunction in CSVD. Considering the differences in cognitive function between the 2 patients, we believe that except for WMH, white matter microstructural changes and multiple brain functional networks cannot be ignored. Changes in brain network connectivity and white matter microarchitecture may be the neuroimaging basis of cognitive decline in patients with WMH and also provide new ideas for the early diagnosis and intervention of CSVD and then improve the quality of life of patients with cognitive impairment. In addition, there are some limitations to this article, and while it is noteworthy that such clinical phenomena were observed in individual patients, these findings are based on case studies and should therefore be interpreted with caution. Further studies, particularly large cohort-based randomized controlled clinical trials, are needed to confirm the results obtained and to generalize our understanding of the association between WMH volume and cognitive dysfunction.

## Author contributions

**Conceptualization:** Hua Zhang, Yuanyuan Wang, Dayong Ma, Chao Zhang, Ruiyun Yu, Haihuan Yang, Xiaocheng Wang, Li Wang, Linjing Song, Jingpei Wei.

**Data curation:** Hua Zhang.

**Methodology:** Hua Zhang, Jingpei Wei.

**Software:** Hua Zhang.

**Supervision:** Hua Zhang, Dayong Ma, Jingpei Wei.

**Visualization:** Hua Zhang.

**Writing—review & editing:** Hua Zhang, Yuanyuan Wang, Dayong Ma, Jingpei Wei.

**Formal analysis:** Yuanyuan Wang, Chao Zhang, Jingpei Wei.

**Validation:** Yuanyuan Wang, Jingpei Wei.

**Writing—original draft:** Yuanyuan Wang.

**Investigation:** Dayong Ma, Ruiyun Yu, Haihuan Yang, Xiaocheng Wang, Li Wang, Linjing Song, Jingpei Wei.
